# Revealing inhibition difference between PFI-2 enantiomers against SETD7 by molecular dynamics simulations, binding free energy calculations and unbinding pathway analysis

**DOI:** 10.1038/srep46547

**Published:** 2017-04-18

**Authors:** Yuzhen Niu, Danfeng Shi, Lanlan Li, Jingyun Guo, Huanxiang Liu, Xiaojun Yao

**Affiliations:** 1 State Key Laboratory of Applied Organic Chemistry and Department of Chemistry, Lanzhou University, Lanzhou 730000, China; 2 School of Pharmacy, Lanzhou University, Lanzhou, 730000, China; 3 Key Lab of Preclinical Study for New Drugs of Gansu Province, Lanzhou University, Lanzhou 730000, China

## Abstract

SETD7 is associated with multiple diseases related signaling pathways. (R)-PFI-2 is the first SETD7 inhibitor with nanomolar inhibitory potency. The activity of (R)-PFI-2 is about 500 times over that of (S)-PFI-2. Understanding the mechanism behind this difference will be helpful to discovery and design of more potent SETD7 inhibitors. A computational study combining molecular dynamics simulation, binding free energy calculations, and residue interaction network (RIN) was performed on the (S)-PFI-2/SETD7 and (R)-PFI-2/SETD7 complexes to explore the molecular mechanism behind the different inhibition activity. The results from Molecular Mechanics/Generalized Born Surface Area (MM/GBSA) calculation show (R)-PFI-2 has lower binding free energy. Residues H252, D256, L267, Y335, G336 and H339 are responsible for the binding of SETD7 to the (R)-PFI-2. RIN analysis indicates van der Waals interaction is critical for the binding of (R)-PFI-2. The results from adaptive basing force (ABF) simulation confirm that the free energy barrier of (R)-PFI-2 dissociating from the SETD7 is larger than that of (S)-PFI-2. (S)-PFI-2 and (R)-PFI-2 dissociate from the SETD7 binding site along different reaction coordinate and have potential mean of force (PMF) depth. Our simulations results will be useful to understand molecular mechanism of activity difference between PFI-2 enantiomers against SETD7.

SETD7 (SET domain-containing lysine methyltransferase 7, also called SET7, SET9, KMT7) functions in transcriptional regulation^1^,^2^,^3^, cell cycle control^4^,^5^,^6^, differentiation^7^, DNA repair^8^ and DNMT1^9^,^10^. Increasing evidences suggest that SETD7 is closely associated with various diseases and. the epigenetic changes induced by SETD7 contribute to vascular dysfunction in patients with type 2 diabetes^11^. As SETD7 is a promising target in several diseases, including diabetes, alopecia areata, cancers and virus infection, several attempts have been made to discovery of SETD7 inhibitors^12^,^13^,^14^,^15^,^16^,^17^,^18^,^19^, but the majority of these inhibitors have weak inhibitory activity. (R)-PFI-2^20^ is a potent and selective inhibitor targeting SETD7 in MCF7 cells. Meanwhile, (R)-PFI-2 shows a much higher inhibiting activity (IC_50_ ≈ 2.0 ± 0.2 nM) with respect to the (S)-PFI-2(IC_50_ ≈ 1.0 ± 0.1 μM). (R)-PFI-2 is the first SETD7 inhibitor with nanomolar inhibitory potency and known mechanism. Therefore, a good understanding of the interaction of each enantiomer with their target protein SETD7 could provide insights to improve their efficacy and is important for designing more potent inhibitors.

Currently, molecular dynamics (MD) combined with binding free energy calculated by Molecular Mechanics/Generalized Born Surface Area (MM/GBSA)^21^,^22^,^23^,^24^ have been successfully used to explore the ligand-receptor interaction. This method can provide not only abundant dynamics structural information on the ligand-SETD7 complex structures in equilibrium phase but also the binding free energy between the ligand and the SETD7 protein. Such information is of importance to understand the detail of ligand-SETD7 interaction and the different inhibitory mechanisms. In addition to the thermodynamics, the binding kinetics between the ligand and the SETD protein is also important to assess the drug efficacy. The adaptive biasing force (ABF) method^25^,^26^ method can markedly improve the accuracy of the free energy calculation, which adds biasing force on the ligand for the purpose of canceling the local barrier acted on the ligand, so the ligand can go with a free-diffusion-like behavior along the reaction coordinate (RC). Residues interaction network (RIN) analysis of the protein-ligand complex can provide some information about the residue interactions to discover possible mechanisms of inhibitory activity. As a result, the combination uses of binding free energy calculations by binding free energy calculation, and network analysis approaches should be effective to understand the inhibition and enantiomer-selectivity mechanism of SETD7.

In our work, we performed a molecular modeling study combining molecular dynamics (MD), MM/GBSA calculations, ABF calculations, and RIN analysis to investigate the mechanism of enantiomer of (S)-PFI-2 and (R)-PFI-2 binding on the SETD7. The MM/GBSA calculations could calculate the binding free energy of the two ligands binding with the SETD7 protein and also identify the key residues for the SETD7 binding to (R)-PFI-2. The RIN analysis could illustrate that the (R)-PFI-2 and (S)-PFI-2 are different in the key interaction residues. The PMF profiles calculated by the ABF could give the information that the difficulty of the two ligands unbinding from the active pocket of the SETD7 protein. Our simulation results show that the higher affinity of the (R)-PFI-2 relative to the (S)-PFI-2 can be related to the different binding mode, binding affinity and different free energy barriers dissociating from the SETD7 binding pocket.

## Materials and Methods

### Preparation of complex systems

**Figure 1:**
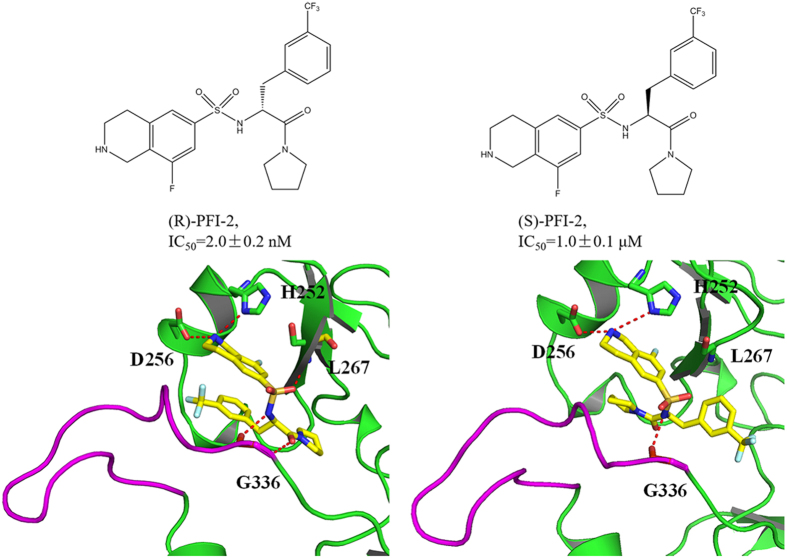
Overview the 2D structure of (R)-PFI-2 and (S)-PFI-2 as well as the binding mode with the SETD7 protein. Left: the (R)-PFI-2 and the binding mode of (R)-PFI-2 with the SETD7; Right: the (S)-PFI-2 and the binding mode of (S)-PFI-2 and SETD7.

The initial atomic co-ordinates for R-PFI-2/SETD7 complex were obtained from the RCSB Protein Data Bank (PDB ID code: 4JLG^20^). The missing residues were fixed and aligned together using Discovery Studio 2.5^27^. We docked the ligand (S)-PFI-2 to the active site of the SETD7 protein by molecular docking to get the structure of (S)-PFI-2/SETD7 complex in Schrödinger 2009^28^ and then the structures of the two complexes were prepared. The 2D structure of the two ligands and the binding mode with the SETD7 protein were shown in Fig. 1. The partial charges of the (S)-PFI-2 and (R)-PFI-2 were calculated at the HF/6-31 G(d) level of theory and fixed using the RESP methodology^29^,^30^,^31^. Each receptor-ligand construct was finally parametrized using the AMBER99SB^32^ and GAFF force fields^33^. Then, the complexes were solvated with TIP3P water models^34^ in a 10 Å cubic box using Leap, and Na^+^ ions were added to neutralize the net charge of the system.

### Conventional MD simulations parameters and protocols

All of the MD simulations were performed with the NAMD 2.9 simulation package^35^. The energy of the two systems was minimized by a steepest-descent minimization scheme for 40000 steps initially and then the temperature of these two systems rose gradually in the NVT ensemble from 0 to 310 K in 100 ps, and during the process a constant force of 10 kcal/mol·Å^2^ was applied to the protein backbone. The restrain decreased from 10 to 0.01 kcal/mol·Å^2^ gradually within 0.9 ns. 100 ns MD simulations were carried out without any restrain. During the whole process of the conventional molecular dynamics (CMD) simulation, the time step, the temperature, the pressure were set 2 fs, 310 K, 1 atm., respectively. The SHAKE^36^ algorithm was used to restrain all bonds involving hydrogen atoms. The cutoff of 10 Å was set to calculate the short-range nonbonded interactions, while the long-range electrostatic interactions was treated by the Particle Mesh Ewald (PME) algorithm^37^.

### MM/GBSA calculations

Although thermodynamic integration (TI)^38^,^39^,^40^ and free energy perturbation (FEP)^41^ are more theoretically rigorous, MM/GBSA^42^,^43^,^44^,^45^ also shows the obvious advantage that the binding free energy can be decomposed into several terms, including the van der Waals, angle, torsion, bond, and electrostatic terms. In MM/GBSA, the binding free energy can be calculated as follows:


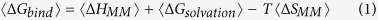


Where 〈Δ*G*_bind_〉 represents the average free energy, and 〈Δ*E*_MM_〉 is the average molecular mechanical energy.


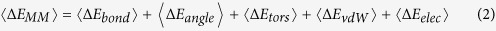



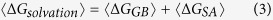


〈Δ*G*_solvation_〉, 〈Δ*G*_*GB*_〉, and 〈Δ*G*_*SA*_〉 are the desolvation free energy upon ligand binding, polar, and nonpolar contributions, respectively. The Generalized Born (GB) model (igb = 2)^46^ is used to calculate the polar contribution of desolvation. The dielectric constants for solvent and solute were set to 80 and 1, respectively. The solvent accessible surface area (SASA) determines the nonpolar contribution of desolvation using the LCPO method^33^:Δ*G*_SA_ = ΔSASA × 0.0072. The normal-mode analysis^47^ is used to calculate the conformational entropy contribution (−*T* 〈Δ*S*〉) in AMBER10^48^.

To determine the contribution of individual residue to the total binding free energy between the two inhibitors and the SETD7, the MM/GBSA binding free energy decomposition process was used to decompose the interaction energy to each residue involved in the interaction by considering molecular mechanics and solvation energy without consideration of the contribution of entropy.

### Residue interaction network calculation

The average structure derived from the last 20 ns MD simulation trajectory of each system was used for constructing the residue interaction network (RIN). The Ring^49^ web server is convenient for identifying of covalent and noncovalent bonds in protein structures, including π-π stacking and π-cation interactions. The software Cytoscape^50^ was used to visualize the residue interaction network with protein residues and their noncovalent interactions represented by nodes and edges, respectively.

### Adaptive biasing force (ABF) simulation

The adaptive biasing force (ABF) method^25^,^51^,^52^ is a powerful tool for determining free energy profile along a chosen reaction coordinate (RC), based on the probability to find the system in the thermodynamic state characterized by a particular value of the reaction coordinate^26^. Therefore, ABF method was employed to study the change of free energy of the two ligands moving out the SETD7 binding pocket along the RC. In details, the reaction coordinate was defined by the distance between the atoms CB of the residue T266 and the atom S of (R)-PFI-2 and (S)-PFI-2. In order to guarantee the direction of the RC, the elastic constant of 5 kcal/mol.Å^2^ was applied to the residues out of 10 Å of the ligand. The details about the parameters setting can refer our previous works^53^,^54^.

## Results and Discussion

### Convergence of the simulation systems

**Figure 2:**
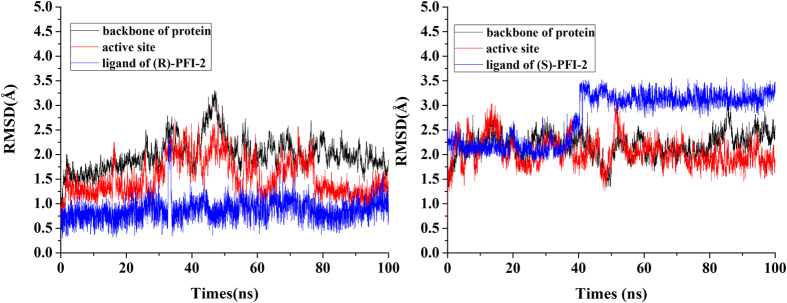
The monitoring of MD trajectories. Left: The (R)-PFI-2/SETD7 complex; Right: The (S)-PFI-2/SETD7 complex. The values reflect the equilibration of each of the systems relative to the initial structures.

MD simulations for the two complexes in solution are run for duration of 100 ns. To explore the dynamic stability of complexes and to ensure the rationality of the sampling method, the root-mean-square (RMS) deviations values of the Cα atoms of the protein, the heavy atoms of the ligand and the Cα atoms within 5 Å of the binding pocket were monitored. As can be seen in Fig. 2, after 50 ns, the RMSD of each system tends to be convergent, indicating the two systems are equilibrated.

**Figure 3:**
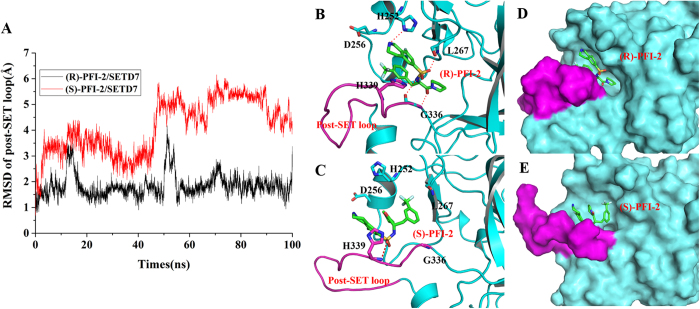
The RMSD fluctuation of the post-SET loop and the average structure of the two complexes from the last 50 ns of CMD trajectories.

We also monitored the RMSD of the post-SET loop (residues 336–349) and the results were shown in Fig. 3. The RMSD value of the post-SET loop of the (S)-PFI-2/SETD7 complex is larger than that of (R)-PFI-2/SETD7complex. In order to analyze the reason behind this conformational change, we obtained the average structures from the equilibrium CMD trajectory (Fig. 3B–E). The detailed binding mode of (S)-PFI-2 and (R)-PFI-2 with the SETD7 protein reveals that the post-SET loop of (R)-PFI-2/SETD7 complex moves out compared with that of the (R)-PFI-2/SETD7 complex,, which. makes (S)-PFI-2 expose more in solvents.

Figure 1 shows that the post-SET loop of the SETD7 exhibits an optimized shape match and forms hydrophobic interaction with the trifluoromethyl moiety of (R)-PFI-2, but this interaction disappears in the (S)-PFI-2/SETD7 complex. The H-bond formed between the residues H252, as well as L267 and G336, and (S)-PFI-2 disappears and the intramolecular π-π stacking interaction between the phenyl group and the droisoquinoline core of the ligand (S)-PFI-2 is also broke up due to the different binding mode of the (S)-PFI-2 with SETD7. The higher inhibitory activity of the (R)-PFI-2 is mainly related to the presence in its complex of a direct H-bond interaction with the residue H252, L267 and G336 while there is only one hydrogen bond interaction between (S)-PFI-2 and the residue H339.

### Binding free energy calculated by the MM/GBSA method

The MM/GBSA method^22^,^55^ has been widely employed to study the ligand and receptor interaction in many cases^56^,^57^,^58^,^59^,^60^,^61^. The calculated binding free energy of (S)-PFI-2 and (R)-PFI-2 binding to SETD7 by the MM/GBSA protocol is shown in Table 1. The calculated contributions favoring to the inhibitors (R)-PFI-2 and (S)-PFI-2 binding include the electrostatic interactions (〈Δ*E*_*ele*_〉), ranging from −374.97 kcal·mol^−1^ for (R)-PFI-2/SETD7 complex to −356.80 kcal·mol^−1^ for (S)-PFI-2/SETD7 complex, and the intermolecular van der Waals energy (〈Δ*E*_*vdw*_〉) ranging from −53.44 to −46.85 kcal·mol^−1^. Nonpolar polar solvation terms (Δ*G*_nonpolar_, are ranging from −60.01 to −53.63 kcal·mol^−1^), which corresponds to the burial of solvent accessible surface area (SASA) upon binding. The polar solvation contribution (Δ*G*_polar_, ranging from 10.20 to 15.67 kcal·mol^−1^) has unfavorable contribution to the (R)-PFI-2 binding. The value of entropic contributions (−TΔS) are 18.58 kcal·mol^−1^ for (R)-PFI-2/SETD7 complex and 15.84 kcal·mol^−1^ for (S)-PFI-2/SETD7 complex, indicating that conformational change of the system is responsible for SETD7-ligand interaction. The total binding free energies (Δ*G*_bind_) predicted for (S)-PFI-2 and (R)-PFI-2 binding with the SETD7 protein are different, with values ranging from −23.23 to −15.84 kcal·mol^−1^. The results prove that the theoretical calculated binding free energies agree with that from experimental values.

### Identification of the key residues for SETD7 binding to (R)-PFI-2

**Figure 4:**
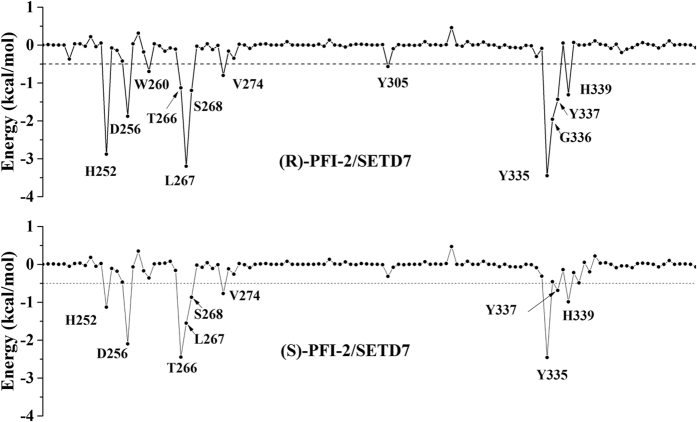
Per-residue decomposition of binding free energy contributions of (R)-PFI-2/SETD7 and (S)-PFI-2/SETD7 complexes.

To obtain a more detailed thermodynamic description of the residue contributions to the binding free energy, we decomposed the enthalpy value (Δ*G*_total,GB_) into a per-residue level depicted in Fig. 4. On the basis of the individual residue contribution to the interaction energy, we identified residues contributing to the binding of (S)-PFI-2 and (R)-PFI-2: H252, D256, W260, T266, L267, S268, Y305, Y335, Y337 and H339 with their contributions varing from −0.57 to −3.45 kcal·mol^−1^ (Fig. 4).

By comparing the individual residue contribution to the binding free energy of (S)-PFI-2/SETD7 and (R)-PFI-2/SETD7 systems (Fig. 4), we analyzed the molecular basis of the difference between the potency changes of (S)-PFI-2 and (R)-PFI-2. We found that the contributions from residues H252, W260, L267, S268, Y305, Y335, Y337 and H339 increased in (R)-PFI-2/SETD7 complex. In contrast, the contributions from residues D256, T266 and G336 decreased. Furthermore, the residues H252 and L267 form hydrogen bond and their contributions decreased in (S)-PFI-2/SETD7 complex. The residues G336, Y337 and H339 are located in the post-SET loop. Their contributions are different in (S)-PFI-2/SETD7 and (R)-PFI-2/SETD7 complexes due to the structure rearrangements of the active site of the (S)-PFI-2/SETD7 complex caused by the conformational change of the post-SET loop. Barsyte-Lovejoy *et al*.^20^ reported the importance of the residues H252, D256 and V274 by site-directed mutagenesis. They conform that the residues H252, D256 and V274 make important contributions to the mode of inhibition by (R)-FPI-2.

### Results from residue interaction network analysis

**Figure 5:**
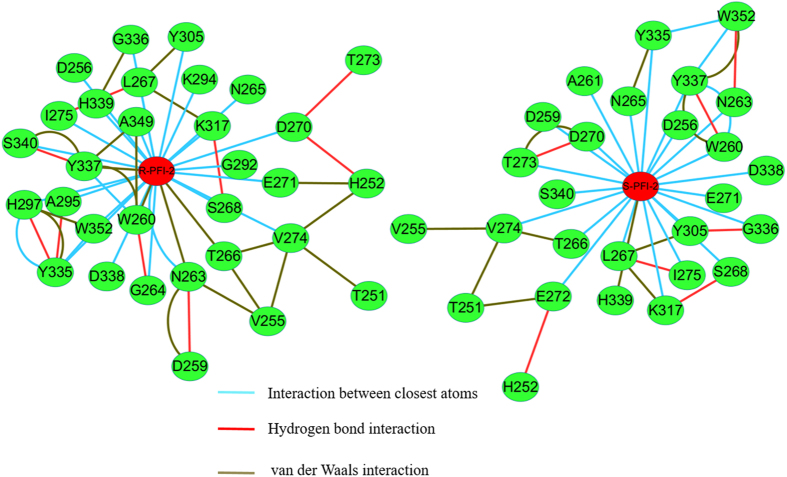
RIN of the (R)-PFI-2 (**A**) and (S)-PFI-2(**B**) binding to the SETD7.

Exploration and analysis of the residues and their ligand interaction network are crucial for understanding protein structure-function relationships^62^,^63^,^64^. Recent studies indicate that exploration and analysis of the network of interacting residues can provide additional insights into the structural and role of residues^65^,^66^,^67^. To explore the binding mechanism of the two ligands with SETD7, we analyzed the information about RIN and the features of the network topologies. In order to clearly explore the interaction between the key residues in the binding site and the ligand, the residues within 5 Å of the ligand were used to generate the representative RIN. In Fig. 5, different types of non-covalent residue interactions including interatomic contacts, hydrogen bonds, and van der Waals overlaps were displayed. The residues W260, L267, Y337 and H339 located in the SETD7 active site could be easily identified as the nodes with the highest number of connections (node degree) in the RIN. (R)-PFI-2 has more connections with its neighboring residues than that of (S)-PFI-2 and connects with the residues N263, T266 and Y337 through van der Waals interaction. (S)-PFI-2 only has the van der Waals interaction with the residue L267. This may explained why the inhibitory potency of (R)-PFI-2 against SETD7 is better than that of (S)-PFI-2.

### PMF calculations and the details of the two ligands dissociation from the SETD7 binding pocket by ABF simulation

**Figure 6:**
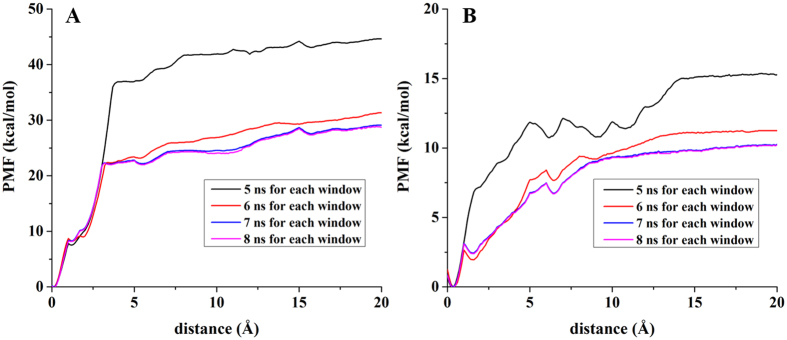
The PMF profiles of the two complexes calculated by ABF. Left: (R)-PFI-2/SETD7 complex; Right: (S)-PFI-2/SETD7 complex. The results show that the PMF is convergent with 8 ns simulation time for each window.

The calculated PMF profiles for (S)-PFI-2/SETD7 and (R)-PFI-2/SETD7 complexes are depicted in Fig. 6. In order to guarantee the convergence of the PMF profiles, we performed the simulation with different times for each window^68^. PMF profiles reach convergent when the simulation time was 8 ns for each window. The free energy curve reveals the information about unbinding of the two ligands. With the departure of the inhibitor from the initial equilibrium position, the free energy value rapidly increases. As can be seen from Fig. 6(A), the initial position of the (R)-PFI-2 is in the most stable binding state of the SETD7. Conversely, in Fig. 6(B), with lower distance, the energy curve sharply decreases. The free energy value rapidly increases with the departure of the inhibitor from the equilibrium position. The free energy barrier (the PMF depth, Δ*G*_PMF,lowest_ − Δ*G*_PMF,highest_) of the inhibitor (R)-PFI-2 unbinding from the SETD7 binding site is around −28.40 kcal mol^−1^ and that of the inhibitor (S)-PFI-2 is around −10.08 kcal mol^−1^. Thus, (R)-PFI-2 needs to overcome a higher energy barrier than (S)-PFI-2 to escape from the SETD7 binding site.

**Figure 7:**
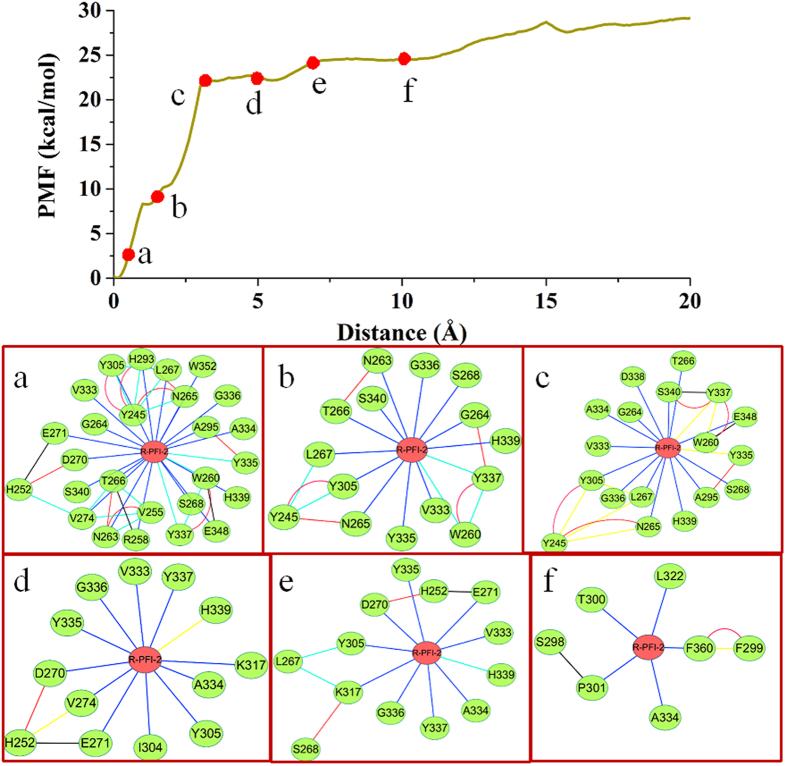
The RIN change of (R)-PFI-2/SETD7 complex along the reaction coordinate in the SETD7 protein. Top: the PMF profile change along the reaction coordinate. Bottom: The corresponding representative RIN of the (R)-PFI-2/SETD7.

**Figure 8:**
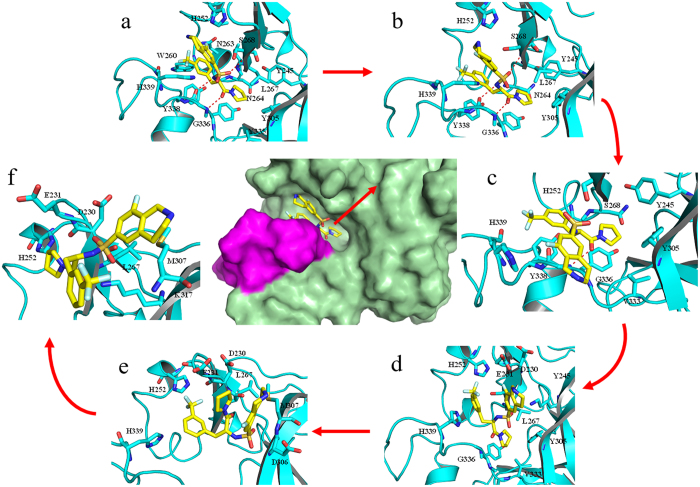
The conformational changes of (R)-PFI-2 along the reaction coordinate.

The above PMF calculation provides important information on energy changes during the unbinding process of the ligand. To explore the atomic essence underlying the energy changes, we carefully investigated the ABF simulation trajectories of the dissociation of the two ligands (S)-PFI-2 and (R)-PFI-2 from SETD7. As shown in Figs 7 and 8, at the beginning the (R)-PFI-2 is the binding site of the SETD7, and the PMF increases until ~3 Å, at this process the system first break up the intramolecular π-stacking interaction between the phenyl group and the droisoquinoline core of the ligand (Figs 7A–C and 8A–C). After ~3 Å, the PMF profile slowly increases and at ~10 Å, the PMF profile shows equilibrium. We could see that the (R)-PFI-2 mainly overcome polar interaction after ~10 Å from Fig. 8D–F.

**Figure 9:**
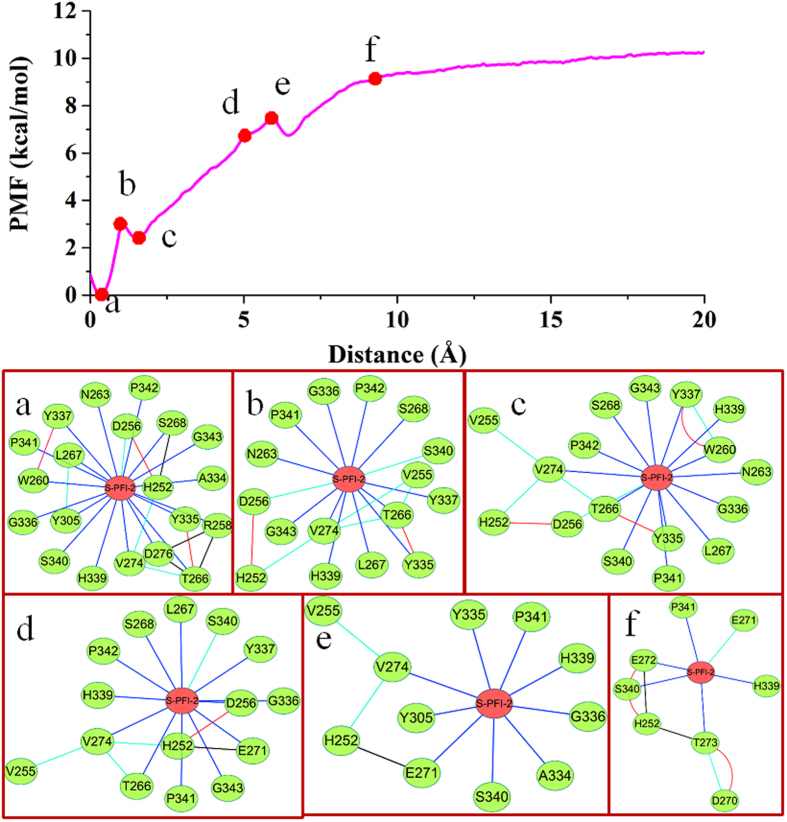
The RIN change of (S)-PFI-2/SETD7 complex along the reaction coordinate in the SETD7 protein. Top: the PMF profile change along the reaction coordinate. Bottom: The corresponding representative RIN of the (S)-PFI-2/SETD7.

**Figure 10:**
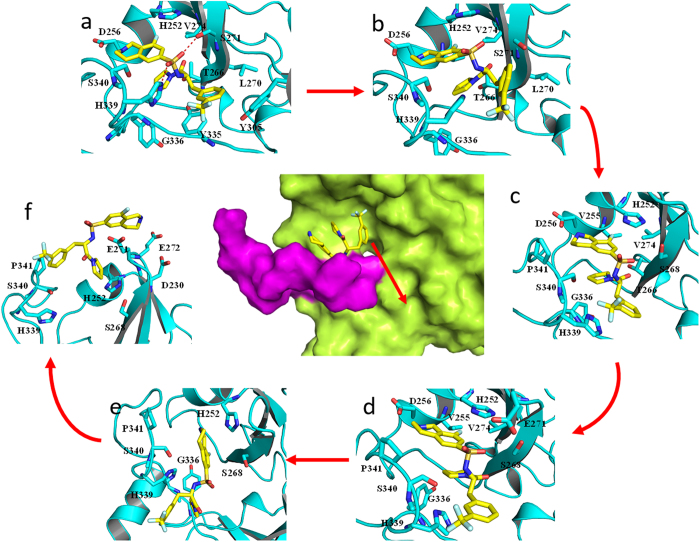
The conformational changes of the (S)-PFI-2 along the reaction coordinate.

It is different for (S)-PFI-2 unbinding from the SETD7 active site, at first the PMF profile decreases (Figs 9A and 10A), and then it increased as the ligand (S)-PFI-2 departure from the SETD7 active site. The PMF profile reaches equilibrium at ~10 Å. The Fig. 10A shows that the reason why the PMF profile decreases at the beginning, is that the stable interaction of the (S)-PFI-2 and the SETD7 due to the post-SET loop movement. As a result, the (R)-PFI-2 and (S)-PFI-2 dissociate from the SETD7 active site along the different reaction coordinate.

## Conclusions

In this work, a computational study combining MD simulation, MM/GBSA calculations and ABF simulation were applied to gain insights into the inhibitory activity differences between two PFI-2 enantiomers against SETD7. The calculated binding free energies predicted by MM/GBSA are in good agreement with the experimental values. The binding free energy decomposition reveals that the binding difference between (R)-PFI-2 and (S)-PFI-2 to SETD7 is mainly from the residues H252, D256, L267, Y335, G336 and H339. RIN analysis illustrates that (R)-PFI-2 has more connections with its neighboring residues than that of (S)-PFI-2. (R)-PFI-2 has van der Waals interaction with the residues N263, T266 and Y337, while (S)-PFI-2 only has the van der Waals interaction with the residue L267.

Analyzing the conformation change of (R)-PFI-2/SETD7 and (S)-PFI-2/SETD7 reveals that the post-SETD7 loop is different in the two complexes. The post-SET7 loop makes the ligand (S)-PFI-2 more exposed to the solvent. Results from residue interaction network analysis in ABF trajectories of the two ligands unbinding from the active site show that (S)-PFI-2 and (R)-PFI-2 have different reaction coordinates. Our computational results clarify why the inhibitory activity of (R)-PFI-2 is better than that of its enantiomer (S)-PFI-2. The results will be helpful to design more potent SETD7 inhibitors. The inhibitors targeting SETD7 should have interactions with the residues H252, L267, G336 and H339, and H-bond between the residues L267, G336.

## Additional Information

**How to cite this article**: Niu, Y. *et al*. Revealing inhibition difference between PFI-2 enantiomers against SETD7 by molecular dynamics simulations, binding free energy calculations and unbinding pathway analysis. *Sci. Rep.* **7**, 46547; doi: 10.1038/srep46547 (2017).

**Publisher's note:** Springer Nature remains neutral with regard to jurisdictional claims in published maps and institutional affiliations.

## Figures and Tables

**Table 1:** Binding free energy between PFI-2 and SETD7 predicted by MM/GBSA method.

Contribution	(R)-PFI-2/SETD7	(S)-PFI-2/SETD7
**Δ*****E***_**ele**_	−374.97 ± 3.67^c^	−356.80 ± 3.81
**Δ*****E***_**vdw**_	−53.44 ± 2.02	−46.85 ± 2.50
**Δ*****G***_**SA**_	−6.57 ± 0.48	−6.78 ± 0.47
**Δ*****G***_**GB**_	385.17 ± 2.22	365.67 ± 2.28
**Δ*****G***_**nonpolar**_^a^	−60.01 ± 4.04	−53.63 ± 2.54
**Δ*****G***_**polar**_^b^	10.20 ± 4.29	15.67 ± 4.44
**Δ*****G***_**total,GB**_	−49.81 ± 5.89	−37.96 ± 5.11
−**TΔS**	18.58 ± 2.18	22.12 ± 3.40
**ΔG**_**bind**_	−23.23 ± 6.28	−15.84 ± 6.14

^a^Δ*G*_nonpolar_ = Δ*E*_vdw_ + Δ*G*_SA._

^b^Δ*G*_polar_ = Δ*E*_ele_ + Δ*G*_GB._

^c^standard deviations calculated through 10 times of repeated sampling from last 50 ns trajectory.
